# Call combination production is linked to the social environment in Western Australian magpies (*Gymnorhina tibicen dorsalis*)

**DOI:** 10.1098/rstb.2023.0198

**Published:** 2024-06-23

**Authors:** Sarah L. Walsh, Simon W. Townsend, Sabrina Engesser, Amanda R. Ridley

**Affiliations:** ^1^ Centre for Evolutionary Biology, School of Biological Sciences, University of Western Australia, Crawley, Western Australia 6008, Australia; ^2^ Department of Evolutionary Anthropology, University of Zurich, Zurich 8032, Switzerland; ^3^ Center for the Interdisciplinary Study of Language Evolution (ISLE), University of Zurich, Zurich 8032, Switzerland; ^4^ Department of Psychology, University of Warwick, Coventry CV4 7AL, UK; ^5^ Department of Biology, University of Copenhagen, Kobenhavn 2100, Denmark

**Keywords:** Western Australian magpies, call combinations, social complexity hypothesis, vocal communication, complex animal signals

## Abstract

It has recently become clear that some language-specific traits previously thought to be unique to humans (such as the capacity to combine sounds) are widespread in the animal kingdom. Despite the increase in studies documenting the presence of call combinations in non-human animals, factors promoting this vocal trait are unclear. One leading hypothesis proposes that communicative complexity co-evolved with social complexity owing to the need to transmit a diversity of information to a wider range of social partners. The Western Australian magpie (*Gymnorhina tibicen dorsalis*) provides a unique model to investigate this proposed link because it is a group-living, vocal learning species that is capable of multi-level combinatoriality (independently produced calls contain vocal segments and comprise combinations). Here, we compare variations in the production of call combinations across magpie groups ranging in size from 2 to 11 birds. We found that callers in larger groups give call combinations: (i) in greater diversity and (ii) more frequently than callers in smaller groups. Significantly, these observations support the hypothesis that combinatorial complexity may be related to social complexity in an open-ended vocal learner, providing an important step in understanding the role that sociality may have played in the development of vocal combinatorial complexity.

This article is part of the theme issue ‘The power of sound: unravelling how acoustic communication shapes group dynamics’.

## 1. Introduction

Recent research has revealed that a diverse range of taxa can combine calls to generate longer, meaningful vocalizations [1–8], collectively indicating that the capacity for combinatoriality may be widespread in the animal kingdom [9]. Despite recent advances in call combinatoriality research in non-human animals, we are yet to understand many of the intricacies involved in the development and production of call combinations, and the factors driving the evolutionary emergence of this complex form of communication remain unclear. Most research to date has focussed on call combinations produced by species with fixed adult repertoires (for reviews, refer [9–13]). For example, the use of call combining in some monkey species [1,4,5,14–17] can expand the number of messages that can be delivered or vary the context-specificity of information contained within messages. Furthermore, in Japanese tits (*Parus minor*) [18] and pied babblers (*Turdoides bicolor*) [2], the meaning of a call combination given during mobbing events is derived from information contained within its two comprising calls (functionally distinct alert and recruit calls), enabling its effective use in a different specific context compared with each comprising call. Previous studies on call combining in animals have proposed that selection favours the combining of calls when there is a constraint to the number of signals (i.e. if the number of messages that can be transmitted is related to repertoire size). However, recent evidence suggests some open-ended vocal learning species (i.e. species capable of continually acquiring/modifying vocalizations through life) may also be capable of combining calls (e.g. some elephant [19], cetacean [20] and songbird [21] species). In light of this evidence, it seems that repertoire size constraint should not be the only factor considered when investigating the potential factors influencing the occurrence of combinatoriality.

One leading hypothesis suggests an evolutionary link between increased communicative capacity and the complexity of the social environment [22,23]. As vocal communication is integral for mediating social interaction and coordinating activities in many social species, having the capacity to communicate a greater diversity of information effectively can provide benefits to individuals living in social groups [24]. Compared with simply structured systems (i.e. where interactions likely occur less frequently and usually between fewer conspecifics), interactions within complex social systems can be frequent, involve a range of contexts and occur between a greater number of conspecifics [25]. There is thus a hypothesized increase in demand for greater and more diverse communicative competence to match the greater complexity of information needing to be transmitted between individuals in more complex social groups (i.e. the Social Complexity Hypothesis for Communicative Complexity: SCHCC) [22,23,26].

Historically, assessing a system’s communicative complexity has primarily been conducted using vocal repertoire size, with greater sociality being positively associated with more signal variability in several taxa [24,27]. For example, one of the earliest studies to show a connection between social and vocal complexity observed that larger and more complex song repertoires were produced by male wrens that occurred at higher densities [28]. However, as changes to a song’s internal structuring do not necessitate any overall change to its informational content [29], song may not be an appropriate conduit for investigations into the SCHCC [24,27]. In contrast, the size of a discrete call repertoire can shed light on the informational content of a vocal system (i.e. transfer of potentially referential information), with call repertoire size being positively correlated with group size in interspecific comparisons regarding primates [30] and bats [31]. These studies suggest that a larger repertoire of distinct signals allows for greater flexibility in the transmission of information. For discrete call repertoire size, however, acoustic variability is generally restricted to a subset of call types and is usually influenced by social function. In comparisons of bird species, for example, high group cohesiveness correlates with the number of contact calls but does not appear to influence the number of alarm calls [32]. However, communicative complexity can also be examined by investigating differences in vocal production activity (i.e. the frequency and diversity of production). Additionally, as complexity in vocal transmission can be increased through the combination of different call types, it is hypothesized that increases in social complexity will positively influence the production of call combinations [33,34].

Crucially, the production of call combinations is associated with social contexts in some taxa [20,35,36], and complexity in the social environment has also been shown to influence the production of combinatorial vocalizations in some cross-species comparisons [8,37]. However, there is currently very little research that considers social influences on intraspecific variation in the production and use of call combinations (but refer ref. [38] for a study showing greater production of combined vocal units related to the social rank of an individual in chimpanzees). Importantly, open-ended vocal learning species may provide unique insight into the factors driving a system to produce combinations despite possessing a capacity to increase communicative output by other means. Furthermore, the ontogeny of call combination production may provide unique insight into the factors promoting this behaviour. Indeed, the effect that the social environment has on vocal development has been studied extensively in song learning [39,40], and within some discrete vocal systems (e.g. [41–43]), but we still know very little about social influences on the ontogeny of non-song combinatoriality, including the potential factors driving its development [44]. Indeed, although open-ended learners continue to acquire and modify vocalizations throughout life [45], auditory-vocal learning is suggested to occur most rapidly during an early vocal development stage in humans and other open-ended vocal learners [46,47]. Thus, it seems prudent to (i) investigate the relationship between social complexity and call combination production and (ii) separately investigate how the social environment may influence call combination production in juveniles compared with adults.

**Figure 1.**
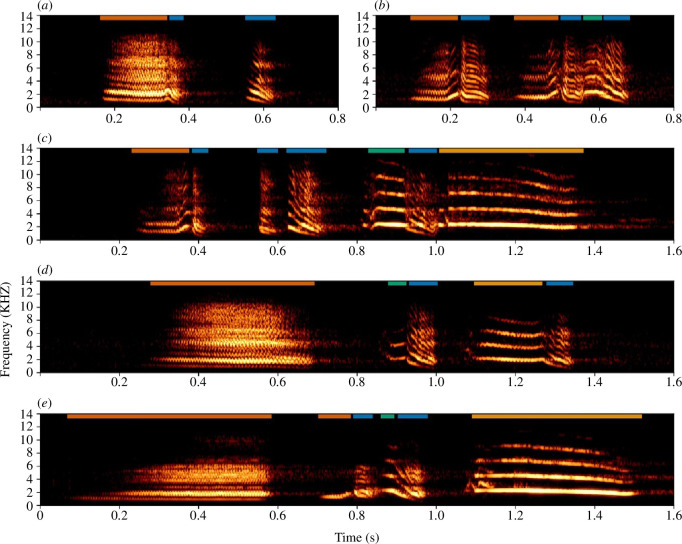
Examples of multi-level combining in magpie vocalizations: segments are combined into calls, which comprise combinations (*a*–*e*). Calls are separated by silent periods and segments are defined by smaller silent periods or sudden spectral shifts. Coloured bars demarcate classes of acoustically similar segments, labelled according to spectrographic appearance; refer to [21] for further detail on magpie combinatorial classification.

Here, we investigate combinatorial production in an open-ended vocal learner: the Western Australian magpie (hereafter, magpie). Magpies are highly social birds that live in stable groups ranging from 2 to 11 individuals and that defend a territory year-round. Group members of all ages and sex interact frequently in a diverse range of contexts [48], such as provisioning young [49,50], antipredator behaviour [51,52] and social bonding through play [53,54]. Vocally, magpies display high communicative flexibility, including a lifelong capacity for learning mimicry [55]. Their vocal system encompasses a range of vocalization types, including a discrete and combinatorial repertoire [21,56] and evidence for urgency coding in alarm calling [52,57]. In previous research, we quantified the magpie combinatorial repertoire (see figure 1 for examples of magpie call combinations), establishing the presence of non-random combining at both the within- and between-call levels. Additionally, we found evidence for ordering rules being shared across separated groups (~17 km) within our study population suggesting combinations may possess some level of functional relevancy in magpies [21]. Magpies are observed to produce distinct types of combinations in a range of social contexts (e.g. recruitment, antipredator behaviour, intragroup competition and territorial defence; [56,58]). Additionally, there is evidence that call combinations can carry information that is meaningful, and that can be perceived distinctly compared with information contained within constituent calls [58]. However, we lack insight into the factors influencing the use of call combinations. Previous research on call combinations during early development in magpies indicates that the rate at which fledglings develop their non-song combinatorial repertoire does not reach a plateau at the point of fledgling independence [59]. Therefore, it appears that the learned acquisition of call combinations is present in magpie fledglings and that it continues beyond this stage (i.e. into the juvenile stage). In light of the vocal complexity present in WA magpies, they present a good opportunity for a detailed examination of the relationship between social and vocal complexity.

To build on our previous classification of the combinatorial repertoire [21], here we focus on the production of call combinations only, with the aim to shed light on the factors potentially influencing their production in magpies. In this study, we integrate natural observation and audio recording to generate a comprehensive dataset for investigating vocal production in juveniles, adults and at the group level. In line with the SCHCC, we predict that callers from larger groups will produce combinations of greater diversity (more distinct combination types) and exhibit greater production frequency of call combinations compared with callers in smaller groups.

## 2. Methods

### (a) Study population

Magpies in southwestern Australia are sexually dichromatic passerines that live year-round in cooperatively breeding groups [50]. Individuals from 12 free-living magpie groups located in the Perth metropolitan suburbs of Crawley (31.97°S, 115.82°E; *n* = 5 groups) and Guildford (31.89°S, 115.97°E; *n* = 7 groups) were observed and recorded for this study. Total group size (adults + juveniles) ranged from 2 to 11 individuals, with a maximum of nine adults and a maximum of three juveniles per group during the study period (see electronic supplementary material, table S1.1 for group data). The groups are part of a long-term research project (founded by A.R.R. in 2014) and are ringed for identification or are otherwise identifiable via distinctive morphological features (e.g. plumage variation or scarring). The studied population has been habituated to human presence, thus allowing for natural observation and audio recording to be conducted at a close distance (i.e. <10 m) [50,60].

### (b) Data collection

Natural observation and audio recordings were collected during weekly group observation sessions between October 2019 and January 2020 and then from October 2020 to March 2021. Over each study period, the time of day at which observation started was randomized between the hours of sunrise and ~12.00 for each group (see electronic supplementary material, table S1.1 for more information), because this is when magpies are most active [61,62]. Observations ceased if air temperature exceeded 31°C, since heat stress is known to impact magpie behaviour at a critical temperature threshold of 32°C [63,64]. Each group observation session lasted 1 h on average (see electronic supplementary material, table S1.1 for a full list of visits to groups). During each group observation session, an observer held a continuously recording microphone and logged all vocal events, noting any contextual information associated with each vocal event (e.g. production associating with a recruitment event or appearance of a predator). For larger groups, up to four researchers were spaced near different individuals throughout the group’s territory, ensuring that as many vocal events as possible could be recorded and allowing later classification of each vocalization.

We originally endeavoured to incorporate individual variation into the analyses, but found this was not feasible during field observations. Athough magpies forage on the ground and can be recorded at a close distance, during one observation period a single individual may fly to perch at a height of up to 30 m above ground level or fly to different locations within their territory. Some territories within our population cover substantial distances, with a maximum territory perimeter of 2.66 km and an area of 0.25 km^2^ (~25 ha). All territories are situated in urban areas, and thus buildings and other structures can hinder the ability to keep a particular bird within line of sight at all times. To be able to identify a vocalization as coming from a specific caller, the researcher needed to be certain that the vocalization could not have come from another group member, or from a bird within a neighbouring group. However, if a focal individual flew to perch high in a tree with other individuals, or to a different location that was out of the researcher’s line of sight, we found that re-identifying a specific individual as the definite caller (based on its rings) was sometimes not possible. Additionally, identification of a single bird at a distance was particularly difficult in larger groups owing to there being a greater potential for misidentification because of the larger number of group members. In comparison to the visual identification of a single bird’s identity, the age and sex of an individual were more easily identified at a distance owing to distinct plumage differences between adults compared with juveniles, and adult males compared with adult females. Thus, to ensure we could obtain a weekly observation for each group and to mitigate the risk of a potential imbalance between smaller and larger groups in the logging of caller-identified vocalizations, we instead focussed our analysis on group-level and age-specific calling behaviour. By having several researchers spread throughout the territory, we were able to collect data on all vocalizations produced within each observation period, resulting in an extensive dataset of call combination production across different-sized groups.

### (c) Data preparation

For this study, we used recordings from a total of 196 observation sessions at 12 different groups, resulting in a cumulative observation duration of ~189 h (average minute duration for weekly observations per group: *x̄* ± s.e. = 58 ± 2.36, range = 55–63; see electronic supplementary material S2; electronic supplementary material, table S2.1). Whole recordings were visually assessed using Adobe Audition (v. 23.5). Each non-song vocal event was classified and labelled according to structure, with labelling reflecting the composition and arrangement of segments comprising the discrete call or combination (see figure 1). We used previously described methods for classification and labelling of vocalizations, whereby discrete calls and call combinations are classified by temporal separation greater than 0.5 s of silence and labels are according to segment structure (see figure 1; for a detailed description of the method, refer to [21]).

To investigate the potential social factors influencing the production of complex vocalizations, we examined variation in the rate of production for (i) call combinations (frequency; averaged by number of calling individuals) and (ii) distinct call combination types (diversity; see below for further clarification on frequency and diversity measurements) across groups that varied in size and composition (e.g. female-to-male ratio, numbers of adults and juveniles). To assess the social factors influencing production during the vocal development stage, we first measured the frequency and diversity of call combinations produced by all juvenile callers per observation (i.e. each group visit). Subsequently, to assess whether the social environment relates to complexity in vocal production at the adult- and group-levels, per observation, we measured the frequency and diversity of call combinations produced by (i) all adult callers only and (ii) all callers within the group.

For frequency analyses, our response variable was the average rate of call combinations for (i) juvenile callers, (ii) adult callers and (iii) all group callers (including vocalizations by unidentified group members), calculated as the number of combinations recorded, averaged by the number of callers per observation session. For example, for a group visit where *N*_CC_ = number of call combinations produced by *N*_I_ number of calling individuals (i.e. either by (i) juvenile, (ii) adult or (iii) all group callers), *F* frequency was calculated as:


$$F=\frac{N_{CC}}{N_{I}}.$$

For analysis of call combination diversity, our response variable was the rate of production for distinct types of combinations produced by (i) juvenile callers, (ii) adult callers and (iii) all group callers. To ensure that diversity measurements do not merely reflect differences in sampling opportunity (i.e. higher diversity simply owing to a greater number of call combinations being observed), we investigated diversity relative to the total number of call combinations recorded. For example, for an observation in which *N*_T_ = number of distinct combination types observed for a total of *N*_CC_ call combinations recorded, *D* diversity was calculated as:


$$D=\frac{N_{T}-1}{N_{CC}}\times 100.$$

Thus, if only one call combination type was observed during the group visit (i.e. *N*_T_ = 1), this would be represented by a diversity rate of 0. In this way, diversity is measured as a rate between 0 and 99, with higher diversity values (i.e. closer to 100) reflecting a larger number of distinct combination types in relation to the number of call combinations that were recorded within observation sessions. To limit inflation in our measurements of diversity, and to be in accordance with our previous investigation [21], we used a simplified labelling system for call combinations that excludes the display of repeated consecutive segments or calls. To limit the potential influence of juveniles on the measurement of diversity at the group level of combination production, we restricted assessment to combinations within the adult repertoire only (i.e. excluding juvenile-only combination types in analysis on group diversity; see electronic supplementary material S2 and electronic supplementary material, figure S2.1).

Accordingly, we independently measured frequency and diversity for (i) juveniles, (ii) adults and (iii) all group callers, which were examined in separate analyses. Out of a total of 196 observation sessions (i.e. weekly group visits), we removed one session (for one group and from all analyses) because combinatorial production during the observation appeared to be impacted by courtship events (specifically, non-normal call arrangement; for further clarification on normal combinatorial arrangement for this species, [21]). To avoid including erroneous counts of zero, respective to the separate (i) adult and (ii) juvenile analyses, an observation session was excluded if call combinations were produced by callers of unknown identity and no other caller could be identified as being either (i) adult or (ii) juvenile (*N*_Adults_: 28 observations; *N*_Juveniles_: 43 observations) [65]. We identified outliers using the package Performance [66] and these were removed prior to each analysis (*n* ≤ 3, per analysis; see electronic supplementary information S2 and electronic supplementary material, figure S2.1 for further detail). For frequency analysis, our final datasets comprised 88 observation sessions for juvenile callers, 164 observation sessions for adult callers and 192 observation sessions for all group callers. For the diversity analysis, our final datasets comprised 87 observation sessions for juvenile callers, 166 observation sessions for adult callers and 194 observation sessions for all group callers [65].

### (d) Statistical analyses

All statistical analyses were performed using generalized linear mixed models (GLMM) with a Poisson distribution and logarithm link function via the package glmmTMB [67] in R (v. 4.2.3) [68]. To explore the potential social factors influencing call combination production, we fitted models with the following predictors: group size (number of adults and juveniles), number of adults (i.e. sexually mature individuals only; magpies generally reach sexual maturity at around 2–3 years), number of juveniles, adult sex ratio (number of females divided by the number of males), year (i.e. austral summer 2019 or 2020) and study site location (Crawley or Guildford). Group identity was included as a random effect in all models to control for pseudoreplication. For analysis on production frequency and diversity, we set observation duration as an offset term to control for variation in the length of observation sessions, log-transformed to match the link function [69]. Initial analyses suggested models were overdispersed (owing to zero inflation), so we attempted analysis using zero-inflated Poisson (ZIP) GLMMs [67,70]. If overdispersion was detected in any ZIP GLMM, we conducted the analysis using a negative binomial model [71,72]. Model assumptions were checked using the packages DHARMa [73] and Performance [66]. Please refer to electronic supplementary information for a full description of model checking for the separate analyses.

### (e) Model selection

Model selection was conducted using Akaike Information Criterion values corrected for small sample size (AICc). Models were compared with a basic model containing only the random term/s. If predictors were highly correlated (according to variance inflation factor (VIF) > 3), the term with the lowest AICc as an individual predictor was used in further additive models [71,74]. The most parsimonious model/s were chosen according to the lowest AICc, and terms within those model/s were considered good predictors of data patterns if their 95% confidence intervals (CIs) did not intersect zero. Where two models had similar AICc values, the model with fewer terms (i.e. simplest structure) was considered more parsimonious [71,75]. The top model set contained all models within 2 ΔAICc of the top model [71] (see electronic supplementary material S2).

## 3. Results

### (a) Call combination frequency

**Figure 2.**
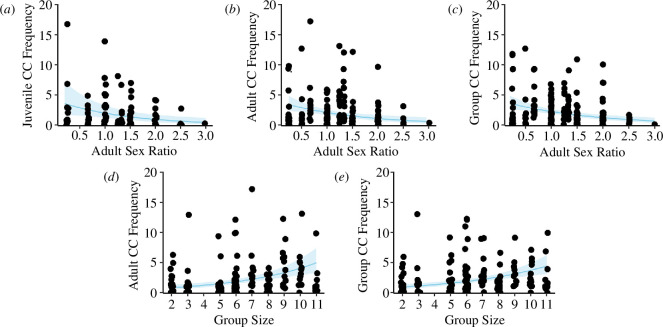
Relationship between (*a*–*c*) adult sex ratio (*N*_Females_/*N*_Males_) and (d–e) group size (*N*_Adults_ + *N*_Juveniles_) with the average frequency of call combinations (CC) produced by juvenile callers (*n* = 88 observations), adult callers (*n* = 164 observations) and all group callers (*n* = 192 observations). Dots denote raw data (rounded average of *N*_CC_/*N*_Callers_ per observation session). Solid lines are trend lines and 95% CIs are indicated by the shaded areas.

The size and composition of the social group positively correlated with the frequency of call combinations produced by (i) juveniles, (ii) adults and (iii) all group callers. Adult sex ratio was the best predictor of the average frequency of call combinations given by juveniles (table 1; electronic supplementary material, table S2.2): juveniles produced combinations at higher frequencies in groups with lower female ratios (figure 2*a*). Significantly, the frequency of call combinations in adults and the whole group (all calling individuals) was also positively affected by both adult sex ratio (figure 2*b*,*c*) and group size (number of adults and juveniles; figure 2*d*,*e*; table 1). Specifically, individuals in larger groups produced combinations more frequently than individuals in smaller groups (table 1; electronic supplementary material, tables S2.3 and S2.4).

**Table 1.** Top model sets for average frequency of combinations produced by: (i) juveniles (*n* = 88 observations), (ii) adults (*n* = 164 observations) and (iii) all group members (*n* = 192 observations). Separate analyses were performed using GLMMs. CI = 95% confidence intervals. Conditional and marginal *r* ^2^ values (*r* ^2^_C/M_), parameter coefficient estimates ± standard error (s.e.) and 95% CIs are provided for each top model set; see electronic supplementary material for further details and full model sets.

	top model set	*r* ^2^_C/M_	AICc	ΔAICc
**(i) juveniles**	adult sex ratio	0.53/0.20	404.95	0.00
	basic intercept		413.11	8.16
**(ii) adults**	group size + adult sex ratio	0.22/0.20	609.55	0.00
	basic intercept		623.79	14.24
**(iii) group**	group size + adult sex ratio	0.19/0.18	717.66	0.00
	basic intercept		732.50	14.84
	**parameter**	**estimate**	**s.e.**	**CI**
**(i) juveniles**	intercept	1.44	0.39	0.66, 2.21
	adult sex ratio	−0.74	0.25	**−1.23, −0.25**
**(ii) adults**	intercept	0.18	0.28	−0.37, 0.74
	group size	0.20	0.04	**0.11, 0.29**
	adult sex ratio	−0.65	0.20	**−1.07, −0.26**
**(iii) group**	intercept	0.34	0.24	−0.13, 0.81
	group size	0.17	0.04	**0.10, 0.25**
	adult sex ratio	−0.59	0.15	**−0.89, −0.30**

### (b) Call combination diversity

**Figure 3.**
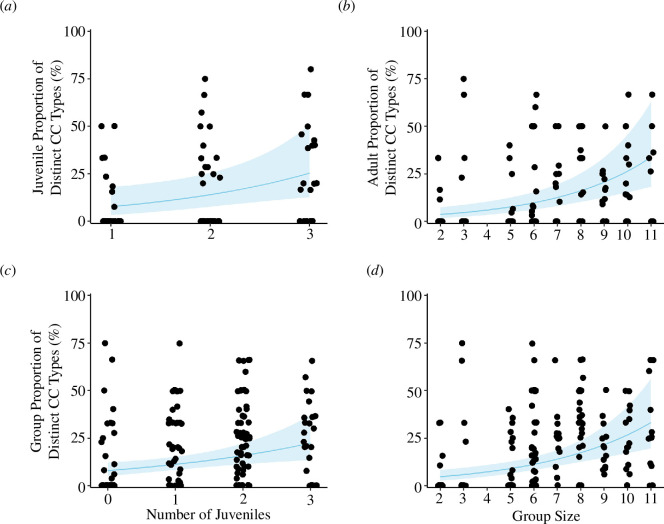
Relationship between (*a*,*c*) number of juveniles, and (*b*,*d*) group size (*N*_Adults_ + *N*_Juveniles_) with diversity for call combinations (CC) produced by (*a*) juvenile callers (*n* = 87 observations), (*b*) adult callers (*n* = 166 observations) and (*c*,*d*) all group callers (*n* = 194 observations). Dots denote raw data (rate of distinct combination types in relation to the total number of call combinations produced within the observation session). Solid lines are trend lines and 95% CIs are indicated by the shaded areas.

Social factors positively associated with the diversity of call combinations produced by: (i) juveniles, (ii) adults and (iii) all group callers. We found the best predictor for juvenile diversity was the number of juveniles within the group (table 2): in groups with more juvenile group members, juvenile callers exhibited a higher diversity of call combinations (figure 3*a*; table 2). Additionally, the number of juveniles correlated with call combination diversity at the whole group level (figure 3*c*; table 2; electronic supplementary material, table S2.5) Finally, the group size positively related to call combination diversity in adults and for all group callers (table 2; electronic supplementary material, tables S2.6–S2.7), with callers in larger groups producing a greater diversity of combinations compared with callers in smaller groups (figure 3*d*).

**Table 2.** Top model sets for call combination diversity rate in (i) juveniles (*n* = 87 observations), (ii) adults (*n* = 166 observations) and (iii) all group members (*n* = 194 observations). Juvenile, adult and group caller analyses were separately conducted using GLMMs. Conditional and marginal *r* ^2^ values (*r* ^2^_C/M_), parameter coefficient estimates ± standard error (s.e.) and 95% CIs are provided for the top model set; see electronic supplementary material for further a detail and full model sets.

	top model set	*r* ^2^_C/M_	AICc	ΔAICc
**(i) juveniles**	number of juveniles	0.10/0.08	490.86	0.00
	basic intercept		494.33	3.47
**(ii) adults**	group size	0.17/0.16	880.61	0.00
	basic intercept		888.42	7.81
**(ii) group**	group size + number of juveniles	0.32/0.30	1300.66	0.00
	basic intercept		1315.88	15.22
	**parameter**	**estimate**	**s.e.**	**CI**
**(i) juveniles**	intercept	−1.47	0.63	−0.23, 2.71
	number of juveniles	0.59	0.25	**0.11, 1.08**
**(ii) adults**	intercept	0.84	0.45	−0.05, 1.73
	group size	0.25	0.06	**0.14, 0.36**
**(ii) group**	intercept	0.81	0.36	0.10, -1.52
	group size	0.22	0.05	**0.11, 0.32**
	number of juveniles	0.34	0.12	**0.10, 0.58**

## 4. Discussion

Our results demonstrate that the production of call combinations in magpies is linked to social factors. Specifically, both call combination diversity and frequency vary according to group size and composition. Importantly, we provide evidence for group size being positively associated with the production of call combinations in magpies, with greater diversity and more frequent emission of call combinations given by individuals in larger groups. In magpies, there is evidence that call combinations can carry information that is meaningful, and they are observed to be produced in a range of social contexts (e.g. recruitment of group members, coordination of mobbing events and territorial defence, as well as antipredator behaviours; [56,58]). Thus, it appears that the findings presented in this study indicate a likely functional relationship between social and communicative complexity in magpies. This finding is in line with other studies that indicate intraspecific variation in vocal complexity relates to differences in social group size and composition [26,38,76,77]. To the best of our knowledge, this study is the first to show that the production of call combinations in a lifelong vocal learning species is correlated with social factors. Crucially, our findings provide support for the SCHCC, which predicts a relationship between social and vocal complexity. As such, our study provides a unique insight into the factors driving the production of call combinations within a system that has the theoretical capacity to otherwise increase communicative capacity (i.e. by learning novel vocal units).

In addition to group size, our findings suggest that other social factors affect the production of call combinations in magpies. Specifically, call combinations are produced more frequently within groups containing a lower ratio of females to males, which is in line with other studies suggesting a link between social organization and vocal complexity [28,37]. For example, Himalayan leaf-nosed bats (*Hipposideros armiger*) in mixed-sex groups produce a larger number of vocalizations, though there is greater complexity in vocal production in groups comprising same-sex individuals [78]. Gu *et al.* [78] suggest that the sex ratio may drive vocal complexity in bats owing to greater social competition between individuals within same-sex groups. In our study, vocal activity may be increased in groups that have lower female-to-male ratios as a means of navigating the more intense social interactions owing to greater competition between males. Indeed, the observation period for our study coincided with the mid–late breeding season, when there are greater rates of aggressive and agonistic interactions [48,79] suggesting that male social competition was present and perhaps influencing vocal activity. Interestingly, previous research suggests that sex differences in magpie singing production may be associated with group adult sex ratio, supporting our finding of a sex ratio effect on vocal behaviour [80]. Thus, it appears there is evidence for greater biases in sex ratio being positively associated with general vocal production; however, the causality in this relationship within magpies remains unclear.

Our study is one of very few to investigate factors that correlate with the production of call combinations in young, with most previous work focussing on adults (but refer [36] for a study showing that the production of call combinations is associated with social contexts in young elephants). However, one might expect that if social or environmental factors are impacting call combination production within a species, the most informative stage to consider these factors would be during vocal development. Indeed, we found that social factors appear to be influencing juveniles during an early stage of vocal development: call combination production varies with the number of other juvenile group members, in addition to the group adult sex ratio. Other studies have found that social interactions with conspecifics can influence vocal development [41,81–83], and our study provides evidence to suggest this is the case for call combination production in magpies. Interestingly, our results suggest that group size does not affect juvenile callers in the same way it does adult callers: group size does not correlate with variation in juvenile call combination frequency or diversity. Our interpretation of a group size–call combination relationship may have been limited by a lack of juvenile data in the smallest groups in our study population (i.e. group sizes 3 and 4), with groups that contain juveniles ranging in size from 5 to 11 individuals. However, we believe the lack of a group size effect is more likely owing to social dynamics in magpie groups. During observation of this species, we observe frequent attacks on juveniles by adults, thus juveniles often form a peripheral sub-unit within the group’s territory. Accordingly, juveniles interact most frequently with other juveniles within these sub-units, thus it is likely the size of their juvenile sub-unit, rather than the total group size, should be considered as the main social influence on their vocal production. Our findings support this notion, with juveniles being more likely to use a greater range of call combinations in exchange with other juveniles compared with in exchanges with adults. Investigating causality in the effect of social interactions on juvenile production of complex vocalizations presents an interesting scope for future study and may shed light on the factors that drive the development of call combinations. Crucially, the effect of social factors has been extensively studied in the development of song [39,40,84]; however, we have much to learn regarding the factors governing the ontogeny of non-song vocalizations [85–87] and very few studies have examined the relationship between social complexity and juvenile call combinatoriality.

Collectively, these findings indicate a relationship between vocal complexity and sociality in magpies, adding to the array of studies [26,30,37,88] that provide support for the SCHCC. Specifically, magpies display greater combinatoriality in groups comprising a greater number of individuals and lower female-to-male ratios. A greater number of individuals and biased sex ratios may increase unpredictability within an environment, potentially resulting in higher levels of social competition and greater diversity in the way individuals interact [22,23,89,90]. Thus, call combining may be a beneficial way to communicate in an unpredictable social environment because it can increase communicative output while reducing the risk of perceptual error [33,91]. Indeed, previous research indicates that vocal diversity and flexibility may increase when there is greater uncertainty in interactions within the social environment [89,90], and we believe this similarly explains the differences in magpie call combination production observed in this study.

In conclusion, we provide evidence in support of the SCHCC by showing that social factors can affect the production of combinations in an open-ended productive vocal learner. We have built on previous work indicating that magpies produce multi-level combinations that are non-randomly arranged [21] and have laid the foundation for future work to delve further into the intricacies of the relationship between social and vocal complexity.

## Data Availability

Analyses reported in this article can be reproduced using the data provided in [65]. Electronic supplementary material is available online [92].
